# Priming primary care providers to engage in evidence-based discussions about cannabis with patients

**DOI:** 10.1186/s13722-019-0171-3

**Published:** 2019-12-02

**Authors:** Devan Kansagara, William C. Becker, Chelsea Ayers, Jeanette M. Tetrault

**Affiliations:** 1 VA Evidence Synthesis Program, VA Portland Healthcare System, Mail Code R&D 71, 3710 SW US Veterans Hospital Road, Portland, OR 97239-2999 USA; 2 Oregon Health & Science University, 3181 SW Sam Jackson Park Rd., Portland, OR 97239 USA; 3 Department of Internal Medicine, Yale School of Medicine, 333 Cedar Street, New Haven, CT 06510 USA; 4 VA Connecticut Healthcare System, West Haven, CT 06516 USA

**Keywords:** Cannabis, Provider-patient relationship, Medical marijuana, Risk mitigation, Mental health, Substance use

## Abstract

Cannabis use has become increasingly common in the U.S. in recent years, with legalization for medical and recreational purposes expanding to more states. With this increase in use and access, providers should be prepared to have more conversations with patients about use. This review provides an overview of cannabis terminology, pharmacology, benefits, harms, and risk mitigation strategies to help providers engage in these discussions with their patients. Current evidence for the medical use of cannabis, cannabis-related diagnoses including cannabis use disorder (CUD) and withdrawal syndromes, and the co-use of opioids and cannabis are discussed. It is crucial that providers have the tools and information they need to deliver consistent, evidence-based assessment, treatment, prevention and harm-reduction, and we offer practical guidance in these areas.

## Introduction

Cannabis use has become more accepted by society over the last two decades, and legalization is now supported by a majority of Americans [1]. Currently 33 states and the District of Columbia (DC) have legalized cannabis for certain medical indications, and in 10 of these states plus DC, it is legal for recreational use [2]. The prevalence of cannabis use has increased in recent years, with past-month use in the United States (U.S.) increasing from 5.8 to 8.4% between 2007 and 2014 [3]. Over 90% of individuals who use cannabis report recreational use, though many patients do not draw a clear distinction between medical and recreational use [4, 5]. The prevalence of frequent cannabis use has also increased over time; from 2002 to 2014, the prevalence of past-year daily cannabis use in the U.S. increased from 1.3 to 2.5% [6]. Increasingly, providers are likely to encounter patients who use cannabis, or who have questions about cannabis use. Thus, in this review article, we aim to provide information about terminology, pharmacology, benefits, harms, and risk mitigation strategies to help providers frame these discussions.

## Terminology, pharmacology, and formulations

The two main classes of compounds in the cannabis plant flower are cannabinoids and terpenes. There are over 100 cannabinoids, but the best studied are delta-9-tetrahydrocannabinol (THC) and cannabidiol (CBD). The effects of cannabis on the body and mind are mediated through the endocannabinoid system, which includes receptors (CB1 and 2) found throughout the body [7]. CB1 receptors are highly concentrated in the brain, though not in the lower brainstem which may explain why cannabis has not been associated with respiratory depression [8, 9]. Most of the psychogenic effect—or the “high”—of cannabis is mediated through the interaction of THC with the CB1 receptor. On the other hand, CBD does not cause potent psychogenic effects due to its low binding affinity for CB1; it also has low binding affinity for CB2. Terpenes have been largely unstudied, but contribute the aroma and flavor of cannabis and are thought to potentially influence the psychogenic effects of the cannabinoids [7]. The term cannabis is typically used when referring to the whole plant or extracts of the whole plant, while the term cannabinoids is used to refer to plant extraction or synthetic products that are isolates of THC, CBD, or a combination of the two.

Cannabis is available and used in many forms. The flower of the plant is the most commonly used form, and there are also numerous plant extract formulations including edibles, resins, oils, tinctures, oromucosal sprays, and topical applications (see Table 1). Two synthetic cannabinoids—dronabinol and nabilone—are FDA-approved for use in chemotherapy induced nausea and vomiting, and AIDS-related cachexia, and one recently-FDA-approved drug, Epidiolex®, used for rare forms of seizure disorders, contains CBD extracted from cannabis plants [10].

**Table 1:** Cannabis formulations and routes of administration

Form	Other terms	Development	Route of administration
Plant	Flower, bud	The highest concentration of cannabinoids are found in the flower, not the leaf, of the female plant	Smoking Vaporization Topical Rectal
Edibles	Brownies, cookies, candy	Typically butter or oil used to extract cannabinoids and put into a variety of edible products	Oral
Tincture	Golden dragon, green dragon	Alcohol or glycerin used to extract active ingredients	Oral Sublingual Oromucosal
Oil		Alcohol used to make highly viscous concentrated extract	Oral Topical
Resin	Hash, dry sift, kief	Concentrate made by mechanically separating trichomes (hair-like protrusions on flower with high concentration of cannabinoids) from the plant	Smoking Vaporization
Dab	Wax, shatter	Ultraconcentrated extract made with solvents such as butane; very high levels of THC, potentially dangerous	Dabbing (concentrate placed on a very metal rod and inhaled)
Nabiximols	Sativex	Pharmaceutically prepared whole plant extract in spray form; 1:1 THC:CBD concentration; approved for use in the EU	Oromucosal
Pharmaceutical cannabinoids	Dronabinol, Nabilone, Epidiolex®	Dronabinol and nabilone are FDA-approved synthetic THC (chemotherapy induced nausea/ vomiting; AIDS related cachexia); Epidiolex® is a highly purified CBD plant extract and is FDA-approved for the treatment of two rare epilepsy syndromes	Oral

There are two main subspecies of the Cannabis sativa plant—Sativa and Indica—though they are largely now indistinguishable because of extensive cross breeding over time [11]. These terms are used colloquially to characterize the expected effects of a given product: Sativa products are purported to have energizing, uplifting, and creative effects (a “mind high”), while Indica strains tend to be sedating, and relaxing physically and mentally (a “body high”). While these terms are commonly used, they are not scientifically grounded. The degree to which a product will have energizing, intoxicating, or relaxing effects is most likely determined by the relative amounts of THC and CBD in the product [3].

There are three main routes of administration: inhalation, ingestion, and topical application (see Table 2 for a summary of the pharmacokinetics and side effects for each) [7]. Smoking and vaping produce rapid peaks in central nervous system THC concentration, and the duration of peak effect is often limited to 30–60 min. “Dabbing,” another method of inhalation, refers to the flash vaporizing of hash oil that is extracted with chemicals like butane and concentrated into a wax or resin that is smoked, produces very high concentrations of THC and an intense intoxicating effect [12]. On the other hand, with the ingestion of edibles (e.g. brownies, cookies, candies, etc.) the peak effect is delayed, often for 2–3 h or more, and the duration of effect is longer. Furthermore, the oral metabolite of THC, 11-hydroxy-THC, is several-fold more potent in its psychogenic effect than THC. Taken together, these characteristics of cannabis edibles explain their role in numerous case reports of acute cannabis-induced psychosis requiring hospitalization after cannabis naïve patients took repeat doses of cannabis edibles because they did not feel an initial effect [13, 14]. Topical applications of cannabis are also widely available but have not been well studied. While they are thought to be associated with minimal systemic absorption and to have lower potential for central nervous system effects, there is little empiric data documenting the potential benefits and harms of topical preparations.

**Table 2:** Routes of administration: compare and contrast

Routes	Smoking	Vaporization (“vaping”)	Oral/edibles	Topical
Notes	Combustion of dried cannabis flower using several methods: cigarettes (joints, spliffs), pipes, water pipes (bongs)	Vaporizer is used to heat dried flower or concentrated extract (oil, resin) and the resultant vapor is inhaled	Variety of edibles available; often dose/single serving is a fraction of the product (i.e.—one part of a cookie or brownie)	Many forms available: creams, ointments, patches, poultices, oils
Pharmacology	Rapid onset and peak so one can gauge effect and titrate dosing	Rapid onset and peak similar to smoking; no smoke; less toxicant exposure than smoking	No inhalation; broad range of products; slower onset action	None of the pulmonary effects associated with inhalation; probably much less intoxicating
Cautions	Bronchial irritation; cough; sputum; production contains carcinogens; potential for adverse effects on lung function with heavy use over many years	Substantially higher blood THC concentrations achieved at a given dose than with smoking; higher risk of adverse effects in novice users; long term lung safety is unknown; need for potentially costly equipment; risk of potentially life-threatening pulmonary illness	Onset and peak are delayed, and effects can last many hours which makes it more difficult to titrate dose; oral metabolite of THC (11-OH-THC) may have fourfold more powerful psychoactive effect risk of overdose; caution especially in novice users	Very little is known about topical preparations; unknown systemic absorption; rapidly expanding topical CBD market is completely unregulated

The potency of cannabis is typically defined as the amount of THC in the product. Given the innumerable formulations and strengths of cannabis available today, it is challenging to define the average potency of cannabis patients will encounter. Moreover, the THC concentration of cannabis has changed over time. For example, the THC concentration of black-market cannabis increased threefold, from 4 to 12%, over the last two decades. Several states have defined a “dose” of THC as 5–10 mg [3]. The average cannabis cigarette (i.e., joint) weighs about 0.32 to 0.66 g [15, 16]. High potency products in dispensaries can contain 15–20% THC, so the amount of THC in a high potency product might be anywhere from 48 to 132 mg. In a recent study of dispensaries in several states, the median amount of THC in a 1.5-g edible product was 54 mg, and the median THC:CBD ratio was 36:1 [17].

Of note, cannabis product labels are often inaccurate. One multi-state study examining THC content of edible products sold in cannabis dispensaries found that only 17% of product labels were accurate when compared to lab measured content [17]. CBD extracts are available over the counter in many locations, as well as online. A study of 84 CBD products purchased online found that only 31% were labeled accurately [18]. In 21% of the CBD products, THC was detected, often in concentrations high enough to produce intoxication, especially in children [18] (which underscores the need to store all cannabis products, including CBD products, out of the reach of children).

### Cannabis for medical use

Medical cannabis laws and implementation of programs vary greatly by state, but in many states with medical cannabis laws, physicians can “certify” that a patient has a condition for which cannabis has been indicated and state-administered programs grant certified patients medical cannabis “cards” [2]. The medical cannabis card then allows patients to enter medical cannabis dispensaries where an employee (often referred to as a “budtender” [19]) discusses the various available products. However, most budtenders have little or no medical training [20]. Most of these states also allow limited home cultivation of cannabis for medical purposes. In states in which cannabis is recreationally legal (all of which also have medical cannabis laws), the medical and recreational dispensaries are often in the same store. Of the states with medical cannabis laws, most have legalized cannabis for the following indications: chronic pain, muscle spasticity and/or multiple sclerosis, epilepsy, anorexia/cachexia, HIV/AIDS, glaucoma, cancer, inflammatory bowel disease, and post-traumatic stress disorder (PTSD) [21]. In a recent nationwide review of state registry data, the most commonly listed qualifying conditions for medical cannabis certification were chronic pain, followed by multiple sclerosis, nausea, cancer, then PTSD [22]. Additionally, one survey study in California found that the most common self-reported indications for cannabis use were anxiety, chronic pain, stress, insomnia, and depression [23].

There are many gaps in the evidence examining the health effects of cannabis, though the literature is expanding rapidly. Many studies have been conducted outside the U.S. in part because of regulatory hurdles associated with the status of cannabis as a Schedule 1 controlled substance at the federal level, which designates it as having “no currently accepted medical use and a high potential for abuse” [24]. A recent National Academy of Sciences report provided a broad and high-level overview of contemporary knowledge about the pharmacology, regulation, benefits, and harms of cannabis for a variety of conditions [3]. It concluded that there is “substantial evidence that cannabis is an effective for chronic pain in adults.” [3]. However, the report did not distinguish the evidence for different types of chronic pain, did not use standard systematic review methods and terminology, and also cautioned that little was known about the efficacy and dosing of cannabis preparations available in the United States.

Two recent systematic reviews that focused on the effects of cannabis for chronic pain found limited evidence that cannabis is effective for neuropathic pain (i.e. pain from peripheral or central nervous system lesions) and for multiple sclerosis-related pain and spasticity in studies of short duration, but insufficient evidence to draw conclusions about the effects of cannabis on nociceptive pain (i.e. pain from tissue damage such as from inflammation, trauma or arthritis) [25, 26]. In placebo-controlled trials of patients with neuropathic pain from a variety of causes, cannabis was associated with a small reduction in pain (about 1 point on a 10-point visual analog scale), and more patients treated with cannabis experienced a 30% or more improvement in neuropathic pain than those assigned to placebo [25]. In patients with multiple sclerosis, cannabis formulations with a combination of THC/CBD in a 1:1 or 2:1 ratio were associated with improvements in pain and spasticity at up to 12–15 weeks of follow up [25, 27]. However, there are numerous limitations to this evidence base. First, findings were not consistent across studies. Second, most studies showing benefit reported outcomes after only a matter of hours to days with the longest study being 15 weeks. Providers should be cautious in extrapolating data from short-term studies to the management of chronic pain, which often requires treatment for many months. Third, the formulations tested differed across studies, and often differed from formulations that are commonly available to, and used by, patients in dispensaries [25]. For example, the best studied formulation of cannabis is nabiximols, a cannabis extract that comes as an oromucosal spray of 2.5 mg THC/2.5 mg CBD; while it is licensed in other parts of the world, it is not available in the U.S. In studies that showed a benefit from nabiximols, participants used an average of 25 mg of THC over 24 h [25].

Oral cannabinoids may improve chemotherapy-induced nausea and vomiting [3]. The synthetic, prescription cannabinoids nabilone and dronabinol appear to be as effective in reducing chemotherapy induced nausea and vomiting as older prescription anti-emetics, but the evidence is limited by methodologic flaws, and not representative of currently available anti-emetics and chemotherapeutic regiments. The authors of one review cautiously suggest cannabinoids may be a reasonable second-line option for patients who have failed or cannot tolerate conventional anti-emetics [28].

There are numerous clinical conditions for which cannabis or cannabinoids have been considered or even approved for use at the state level despite a lack of controlled studies in many cases. There is largely insufficient evidence to support or refute the effectiveness of cannabis and/or cannabinoids for the following conditions: irritable bowel syndrome, amyotrophic lateral sclerosis, Parkinson’s disease, dystonia, post-traumatic stress disorder, and glaucoma. There are very few high-quality studies examining the effects of cannabis and oral cannabinoids on weight loss and anorexia associated with HIV/AIDS and cancer. While there is insufficient evidence for the effectiveness of cannabis or cannabinoids for most forms of epilepsy, the FDA recently approved a highly purified CBD plant extract called Epidiolex® for the treatment of two rare childhood-onset epilepsy syndromes on the basis of trials showing significant reduction in seizure frequency [29, 30]. There has been increased interest in additional potential therapeutic effects of CBD alone, in part because it is viewed as likely to have a more favorable adverse effect profile than THC-containing products. While there is not conclusive evidence supporting the health effects of CBD preparations beyond the epilepsy syndromes mentioned above, a growing body of evidence suggests CBD may have potential as an anxiolytic agent, though further research is needed [31, 32].

### Potential harms of cannabis use

#### Cannabis and other substance use disorders

Cannabis use is associated with the development of a cannabis use disorder (CUD), characterized by craving, tolerance, withdrawal, and continued use despite adverse social, vocational, or legal consequences of use (see Table 3) [33]. Authors examining data from multiple waves of the National Epidemiologic Survey on Alcohol and Related Conditions (NESARC) sought to determine the association of cannabis use with subsequent mental health and substance use disorders and found that among survey participants with past-year cannabis use, 36% met criteria for CUD; among those reporting cannabis use in wave 1, the incidence of CUD 7 to 8 years later was 25% [34]. Nearly half of those with CUD had moderate to severe disease, and men, low-income participants, adolescents and young adults were at highest risk of developing CUD [35]. Indeed, the risk of CUD was up to 50% greater among individuals who used daily and initiated use in adolescence [34, 36].

**Table 3:** Stepwise assessment of cannabis use disorder

Step 1. Do you currently use cannabis? YES/NO				
Step 2. IF YES, cannabis use disorder-short form [78]				
1. How often during the past 6 months did you find that you were not able to stop using cannabis once you had started?				
Never	Less than monthly	Monthly	Weekly	Daily or almost daily
0	1	2	3	4
2. How often in the past 6 months have you devoted a great deal of your time to getting, using, or recovering from cannabis?				
Never	Less than monthly	Monthly	Weekly	Daily or almost daily
0	1	2	3	4
3. How often in the past 6 months have you had a problem with your memory or concentration after using cannabis?				
Never	Less than monthly	Monthly	Weekly	Daily or almost daily
0	1	2	3	4
Total score _______				
Positive Screen = 2 or higher				
Step 3: Confirm with DSM-V Criteria for Cannabis Use Disorder [36]				

Cannabis withdrawal is now considered a Diagnostic and Statistical Manual (DSM)-5 syndrome and is associated with symptoms that may develop up to 1 week after cannabis cessation and last up to several weeks [33]. Many of the symptoms of cannabis withdrawal such as anxiety, depression and insomnia overlap with symptoms that patients may be using cannabis to alleviate in the first place, which underscores the need for the public and providers to be aware of this withdrawal syndrome.

Cannabis use is not only associated with increased risk of CUD, but also with a sixfold increase in risk of developing any substance use disorder (SUD) [34]. From an emerging body of evidence with mixed findings it is unclear whether those using cannabis only for medical purposes have a different risk of CUD and SUD from those using for recreational purposes [37, 38].

#### Other mental health harms

Substantial evidence suggests an association between cannabis use and the development of psychotic symptoms, with increasing risk among individuals with more frequent cannabis use [25, 39]. Consumption of a large amount of THC can cause acute psychosis, but there is also data that regular cannabis use—especially in young adults and among those with a genetic predisposition to schizophrenia—is associated with the development of chronic psychosis [39, 40]. It is unclear whether cannabis use is associated with other mental health outcomes such as suicidal ideation, depression, anxiety, and mania [3, 25].

A large and growing body of evidence demonstrates an association between active, long-term cannabis use and small but significant negative effects on all domains of cognitive function, though it is unclear whether cognitive deficits persist in those who have stopped using cannabis [25, 39]. Concerns for cognitive dysfunction are magnified in adolescents with cannabis use because brain development continues into young adulthood. Findings from a birth cohort study suggested that ongoing cannabis use during adolescence is associated with neuropsychological decline broadly across domains of functioning, even after controlling for years of education. The study also found that cessation of cannabis use did not fully attenuate cognitive decline in adulthood [41]. Other studies focus more on the acute neuropsychological implications of cannabis use. The National Institute of Health (NIH) is currently embarking on a large population study, The Adolescent Brain Cognitive Development (ABCD) Study, which will add significantly to the evidence base in this area [42].

#### Physical health harms

The literature does not show a direct association between prolonged cannabis use and measures of airflow obstruction or development of lung cancer [25]. There are data suggesting respiratory harms in those with frequent smoked cannabis use (i.e. more than weekly), in older adults, and those with medical comorbidities [25]. A few large epidemiologic studies suggest that prolonged daily cannabis smoking may be associated with a decline in lung function over two decades, and systematic reviews note an association between cannabis smoking and symptoms of chronic bronchitis [3, 43, 44]. An emerging case series of severe pulmonary illness associated with inhalation of vaporized products typically bought illicitly, and often containing THC, has recently been described [45]. The cause is unclear, but is thought to be related to chemicals in vaping products or equipment, [45] and underscores the uncertainty and potential danger of vaporizing unregulated cannabis products.

While cannabis can increase heart rate, supine blood pressure, and postural hypotension, there is insufficient evidence to draw conclusions about its effects on cardiovascular health outcomes [46, 47]. There is likewise insufficient evidence examining effects on most types of cancer [25].

Driving under the influence of cannabis poses significant public health risks. Systematic reviews and meta-analyses suggest a low-to-moderate association between cannabis use and motor vehicle crashes, even when accounting for alcohol or other substance use [48, 49]. Most of the studies included in these reviews are case–control studies or culpability studies; therefore, more research is needed to understand the complex relationship between driving under the influence of cannabis and vehicular injuries especially as more states enact recreational cannabis legislation.

Cannabis hyperemesis syndrome is a form of cyclic vomiting that is thought to be associated with chronic, regular cannabis use [50]. Most patients also present with abdominal pain and many patients report improvement of symptoms with hot showers [50]. The treatment is discontinuation of cannabis, though improvement may take many weeks and the effectiveness of abstinence has not been well studied [50]. There are a variety of case series suggesting potential efficacy for a variety of pharmacotherapies, though there are no trial data to guide treatment choice [51].

Another concern regarding recreational cannabis laws is the risk of pediatric exposure and overdose, especially related to edible products. In states that have enacted medical and recreational cannabis laws, the absolute number of hospitalizations and poison control center calls are low, but the rates of unintentional pediatric exposures continue to increase compared with states that have not enacted these laws [52-54]. This should prompt policymakers to pass more stringent regulations regarding cannabis product packaging, labeling and marketing.

#### Synthetic cannabinoid harms

Synthetic cannabinoids are man-made cannabis derivatives often marketed as safe, legal alternatives especially in states without recreational cannabis laws. The route of administration is most often inhaled, but they can be ingested as well. They are often substantially more potent CB1 receptor agonists than plant-derived THC [55]. Clinical effects may be similar to intoxication with cannabis but may present more acutely and include tachycardia, vomiting, ataxia, violent behavior, suicidal ideation, sedation, and slurred speech. There have been case reports and case series of “outbreaks” of mass intoxication with synthetic cannabinoids such as FUBINACA and K2 [55-57].

### Cannabis and opioids

Pain is a leading symptom driving non-recreational cannabis use, whether medically authorized or not. Despite recent efforts to reduce prescribed opioid therapy for pain in the U.S., and to increase treatment for opioid use disorder (OUD), opioids remain a prominent treatment for both acute and chronic pain, and untreated illicit opioid use has reached epidemic levels [58]. Given widespread opioid and cannabis use, both illegal and prescribed, cannabis and opioid co-use is increasingly common.

#### Potential benefits of co-use, opioid sparing effects

Widely-cited ecological studies have demonstrated an association between states allowing medical and/or recreational cannabis use and declining opioid prescription [59]. OUD hospitalization [60] and overdose rates [61] driving a hypothesis that opioid users may be substituting cannabis and decreasing risky opioid use. However, a more recent analysis demonstrated increased rates of opioid overdose death among states with more liberal cannabis laws, including those permitting recreational cannabis use [62]. At the individual level, there are studies suggesting that cannabis augments the analgesia produced by opioids, [63, 64] potentially allowing opioid dose-lowering, thus possibly enhancing safety. One cross-sectional study of 244 individuals with chronic pain who were receiving cannabis from a dispensary found that these individuals reported decreased opioid use over time because of the benefits of cannabis for pain [65]. An ongoing online survey of 1321 participants, found 53% reported substituting cannabis for opioids, citing fewer side effects and better symptom management as their rationale for doing so [66]. Prospective, individual-level studies are needed to examine cannabis’ potential role as an opioid sparing agent.

#### Potential harms of co-use

With respect to overdose, the most common opioid-related cause of death, there is a dearth of data on the potential additive risk of cannabis co-use [67]. As for non-overdose harms, the synergistic effects on psycho-motor slowing, depressed sensorium, and delirium (neurocognitive effects) of co-use may lead to increased risk of motor vehicle accidents, falls, and trauma, all known dose-dependent risks of opioids by themselves [67, 68]; more epidemiologic studies are needed. With respect to concerns of association with SUD, observational data suggest that cannabis use is associated with opioid misuse among patients on long-term opioid therapy [69] with a more recent study demonstrating nearly sixfold increased odds of non-medical opioid use among those with cannabis use [70].

A practical challenge that can lead to unintended harm in co-use of cannabis and opioids is that opioid prescribing guidelines typically recommend a single prescriber (or team) who makes treatment decisions with the patient [71, 72]. In practice, a provider who offers medical cannabis certification is likely to be someone other than the opioid prescriber, giving rise to potential conflicts in treatment philosophies. The opioid prescriber’s assessment of potential benefit and risk may differ from the cannabis certifier and the prescribers sometimes abruptly discontinue opioid therapy with evidence of cannabis use, whether certified or not [73, 74]. Finally, issues of provider liability may become complex if a patient who has both an opioid prescriber and a medical cannabis certifier were to overdose.

#### Cannabis for treatment of OUD

Recently, some states have introduced the treatment of OUD as a medical indication for cannabis [75]. However, there is very little research examining the effectiveness of cannabis for this indication. Moreover, there are three evidence-based medication treatments for OUD—methadone, buprenorphine and naltrexone—each of which have robust data from randomized controlled trials (RCTs) demonstrating improvement in important patient- and public-health outcomes [14]. Given the existence of evidence-based therapies for OUD, the lack of research examining cannabis for this indication, and the potential harms we agree with other commentators that the use of cannabis for this indication is premature [75].

### Provider advice for individuals using cannabis

There is currently not enough evidence to suggest that the long-term benefits of cannabis are likely to outweigh the harms, though our understanding of the balance of benefits and harms of cannabis is very likely to change as research accumulates. While there is no conclusive evidence to support providers’ endorsement of widespread medicinal use of cannabis, the reality of clinical practice today is that patients have access to and are using cannabis, and it is the provider’s duty to play a role in reducing any likelihood of harm.

When asking patients about cannabis use, it is important to do so in a routine, non-judgmental fashion, and to ask about it separately from illicit drug history because cannabis is legal in many parts of the country. The provider may also want to ask about the reasons for use—whether recreational, medical, or both—because the reason for use may influence its frequency and route of administration; for medical use, this also allows for follow-up to assess whether or not use for medical purposes is having the intended effect [76, 77]. When a provider encounters a patient who uses cannabis, the first step is to determine the quantity, frequency, and route of administration, as well as assessing for CUD. There are no widely validated short CUD screeners, but one option is use of the three-item Cannabis Use Disorders-Short Form to assess for CUD [78], and if the results are positive the provider should follow up by assessing for CUD with DSM-5 criteria (see Table 3).

Treatment of CUD should include evidence-based behavioral therapies, such as cognitive-behavioral therapy and motivational enhancement therapy along with abstinence-based incentives, targeted at cannabis cessation [79]. While a number of pharmacotherapies for CUD have been evaluated—including gabapentin, N-acetylcysteine, antidepressants, and cannabinoids—the evidence is currently not strong enough to support routine use of pharmacotherapy for CUD [80]. For patients who use cannabis, but do not meet criteria for CUD, providers should counsel them on potential harms and provide psychoeducation and behavioral support.

Given the risk of withdrawal with CUD, providers should counsel patients who continue to use cannabis to avoid frequent, heavy use. Indeed, simply outlining the symptoms of withdrawal such as depression, anxiety, insomnia, and restlessness may help patients avoid a dangerous cycle of self-medication for withdrawal symptoms. It is likely that THC in particular is responsible for many of the potential mental health harms, so providers should suggest that patients avoid high-THC products. Patients at risk for mental illness, especially psychotic spectrum disorders, should be counseled to avoid cannabis.

The formulation of cannabis and route of administration influence its potential adverse effects. Patients should be made aware of the potent metabolite and the delayed onset of action associated with edible products, and accordingly use low doses, and avoid rapid dose escalation. Cannabis naïve patients should be especially cautious with edible products, and all individuals should avoid dabbing altogether given the high potency and risk for adverse effects. Individuals should avoid frequent and long-term cannabis smoking, and long, deep breath holds during inhalation. Providers should warn against use of any unregulated product obtained outside of dispensaries, including products advertised as CBD-only, given the existence of dangerous synthetic cannabinoids, labeling inaccuracies, and the risk of severe illness such as vaping-related pulmonary illness.

For patients who are prescribed other central nervous system (CNS)-acting agents, including, for example opioids, benzodiazepines, muscle relaxants, and gabapentinoids, we would encourage prescribers of those medications to caution patients that the additive effect of cannabis on psychomotor slowing and other CNS side effects has not been well studied. We recommend a conservative approach whereby providers decrease CNS-acting agent polypharmacy (in safe, patient-centered ways) prior to or concomitant with patients initiating cannabis regimens.

### Providers’ responsibility to public health

With the changing landscape of cannabis legislation throughout the U.S., and more states legalizing recreational cannabis, medical providers need to mindfully frame discussions of cannabis use with patients and families. To counter the inaccurate public perception of cannabis as harmless, the medical community must be prepared to synthesize the evidence and deliver clear public messaging on the potential harms of cannabis use. Current evidence suggests that recreational cannabis use is associated with other substance use and SUDs; mental health conditions; impairment in memory, learning and attention; respiratory symptoms; motor vehicle crashes; and overdose injuries in pediatric populations [3, 25, 81]. Conversely, advocates of legalization argue that the criminalization of cannabis has had substantial social justice and public health implications through contact of millions of Americans with the criminal justice system [82]. There were 8.2 million cannabis-related arrests from 2001 to 2010, with African Americans four times more likely to be arrested; most of these arrests were for simple possession [83]. The downstream effects of these arrests can be detrimental as the existence of a criminal record can act as barrier to employment, housing, and public services for those arrested [83]. Ultimately, legalization will help to mitigate some of these structural inequities. However, legalization must be accompanied by a concerted public health effort to counter the potential harms of cannabis use.

This is particularly important for adolescent and young adult populations because we know most harms are related to use of smoked cannabis products among individuals who began smoking in a heavy, habitual way in adolescence. Development of prevention messages geared to adolescents and young adults is vital. Data from the Monitoring the Future study, which is an annual survey of 8th, 10th and 12th grade students in the U.S., suggests that for three decades there was an inverse relationship between perceived risk of cannabis and cannabis use among high school students. Over the last few years, perceived risk has continued to decline steeply but there has not been a concomitant further rise in use, creating an opportunity for the medical community to focus efforts on effective prevention interventions [84].

## Conclusions

The changing landscape of cannabis legislation throughout the U.S. has had an impact on the prevalence and perceptions of safety and harms of cannabis use. With increasing frequency, patients are turning to providers to inquire about a full spectrum of cannabis effects: from medical use to potential harms. Given the widespread use of cannabis to manage symptoms of chronic pain at the same time as the country is facing an opioid epidemic, it is not uncommon for patients to have questions about use of cannabis and opioids for pain. It is imperative that primary care providers understand the evidence to engage in balanced discussions with patients. Additionally, primary care providers need to have tools to assess for problem cannabis use and have a mechanism for referring patients for treatment if indicated. It is crucial that the medical community deliver consistent, evidence-based assessment, treatment, prevention and harm-reduction messaging to dispel myths and prevent potential public health problems related to cannabis use.

## Data Availability

Not applicable.

## Abbreviations

ABCD: adolescent Brain Cognitive Development Study
AIDS: acquired immunodeficiency syndrome;
CBD: cannabidiol
CUD: cannabis use disorder
DC: district of Columbia
DSM: diagnostic and Statistical Manual
ED: emergency department
FDA: food and Drug Administration
HIV: human immunodeficiency virus
NESARC: national Epidemiologic Survey on Alcohol and Related Conditions
NIH: national Institutes of Health
OUD: opioid use disorder
PTSD: post-traumatic stress disorder
RCT: randomized controlled trial
SUD: substance use disorder
THC: delta-9-tetrahydrocannabinol
U.S.: united States
